# Quantification of Free Circulating DNA and Differential Methylation Profiling of Selected Genes as Novel Non-Invasive Biomarkers for Endometriosis Diagnosis

**DOI:** 10.3390/biom15010069

**Published:** 2025-01-06

**Authors:** Moncef Benkhalifa, Pierre Alain Menoud, David Piquemal, Jack Y. Hazout, Sami Mahjoub, Mohammed Zarquaoui, Noureddine Louanjli, Rosalie Cabry, Andre Hazout

**Affiliations:** 1 IVF & Reproductive Genetics and PeRiTox Laboratory, University Hospital & School of Medicine, Picardy University Jules Verne, 80000 Amiens, France; cabry.rosalie@chu-amiens.fr; 2 Unilabs, Molecular Diagnostic Laboratory—Lausanne, CH-1011 Lausanne, Switzerland; pamenoud@gmail.com; 3 ACOBIOM, Biopole Euromédecine II Montpellier, 1682 Rue de la Valsière, 34790 Grabels, France; piquemal@acobiom.com; 4 ENDOLIFE, 28 Rue de Courcelles, 75008 Paris, France; jack.hazout@icloud.com (J.Y.H.); hazout.andre@hotmail.fr (A.H.); 5 Alyssa Fertility Group (AFG), Clinique Alyssa, Tunis 1053, Tunisia; docteursamimahjoub@yahoo.fr; 6 IRIFIV Center, Les IRIS Clinic, Casablanca 20000, Morocco; mozar92@gmail.com (M.Z.); n.louanjli@gmail.com (N.L.)

**Keywords:** endometriosis, free circulating DNA, methylation profiling, non-invasive test

## Abstract

Endometriosis is a chronic, estrogen-dependent disorder associated with the presence of endometrial cells mainly in the pelvic cavity, causing systemic immune inflammation, infertility, epigenetic dysregulation of differential DNA methylation, coelomic metaplasia, and pain. It affects approximately 10–12% of women. Despite decades of research, full pathophysiology, a diagnostic roadmap, and clinical management strategies for endometriosis are not yet fully elucidated. Cell-free DNA (Cf-DNA) in the peripheral blood of diseased and healthy individuals was discovered in the 1950s. Quantifying peripheral Cf-DNA and the specific differential methylation of a group of genes have been proposed as potential non-invasive diagnostic biomarkers for somatic and constitutional genetics and for various other pathological disorders. In this study, we investigated the Cf-DNA levels of 78 young women, 38 of whom had endometriosis confirmed via laparoscopy and 40 of whom were healthy. We found a significant difference between the two groups when Cf-DNA was quantified, with 3.9 times more Cf-DNA in the serum of women with endometriosis. We also identified nine target genes potentially involved in the pathogenesis of endometriosis, with a different methylation profile between the two groups. Our data suggest that the combination of cell-free DNA quantification and the assessment of the epigenetic signature of differential methylation of nine genes can be proposed as a non-invasive predictive and diagnostic test for endometriosis.

## 1. Introduction

Endometriosis has become a global health problem, affecting nearly 10–12% of women. This pathology is a systemic, estrogen-dependent immunoinflammatory syndrome often combined with epigenetic DNA differential methylation dysregulations that occur after the migration of endometrial cells outside the uterine cavity, mainly into the organs and tissues of the pelvis. Endometriosis can originate in situ from local embryological tissues that can develop during organ development of the fetus in the mother’s womb or via cellular metaplasia through the transformation of differentiated cellular tissue into another tissue, such as endometrial cysts, without knowing the cause [1,2,3,4]. This pathology is associated with pelvic pain and infertility in nearly 20% of cases [5] and can also occur at prepubertal age, accompanied by severe pain [6], or between puberty and menopause. It is mainly associated with the recirculation of menstrual blood via the migration of endometrial cells to the surrounding organs and sometimes beyond (the abdomen, lungs, brain, and elsewhere) [7]. Endometriosis has also been found in the male genitourinary tract [8].

Endometriosis is a heterogeneous disease in terms of symptoms, response to treatment, and the presentation of endometriotic lesions ranging from superficial and sub-peritoneal lesions to endometriotic ovarian cysts (endometriomas), deep endometriosis with nodules, adhesions, and fibrosis. Epidemiological studies have reported that sporadic irregular menstrual cycle dysregulation can affect 76–90% of women of reproductive age [5], but only 10–12% of women have endometriosis. It has been reported that endometriosis may be caused by hormonal or immunological dysregulation and/or possible dynamic genomic and epigenomic variation caused by occupational and/or environmental exposure to endocrine disruptors and lifestyle changes [9,10]. Several major studies have led to the identification of genetic variants significantly associated with the disease [10]. However, these genetic causes explain less than 10% of cases of endometriosis. Current evidence suggests the conversion of uterine stem cells into tumor-initiating stem cells in endometriosis, as well as adenomyosis, acquiring genetic and epigenetic alterations [11,12].

In patients who are symptomatic, classical methods of investigation have revealed a large gap between the heterogeneity of the disease phenotype and laparoscopy results by demonstrating that anatomopathological, immunohistochemical, and epigenetic studies of lesions are unreliable. A dysfunction in the immune system is also suspected because of the various alterations already being described: chronic inflammation, increased presence of regulatory T lymphocytes, and the possible alteration of macrophages, which play a role in the appearance of lesions [13]. However, research is not yet mature enough to know whether this is a factor that promotes the development of endometriosis or a consequence of the disease.

By affecting nearly 12% of the female population worldwide, endometriosis is considered a public health problem impacting the financial costs of medical management and patients’ quality of life and well-being [14]. In current medical practice, only patients presenting with symptoms are diagnosed after clinical examination, followed by an imaging work-up, including pelvic ultrasound and sometimes pelvic MRI.

When endometriosis is suspected based on clinical symptoms, a small number of patients are referred to a surgeon for laparoscopy with biopsy and cytological analysis to confirm the diagnosis [15,16].

It has been reported that ectopic endometrium has a distinctive epigenetic expression profile, which involves homeobox A (HOXA) and Wnt signaling pathway genes [17]. Furthermore, the known role of extra-cellular vesicle miRNA dysregulations was found to modulate the proliferation and invasiveness of ectopic endometrial cells [18].

In clinical medicine, cell-free DNA (Cf-DNA) is starting to be considered a promising biomarker for prognostics, monitoring disease response, the detection of minimal residual disease, and early diagnosis [19]; it has also been reported as a biomarker in endometriosis [20,21]. In 2022, Bendifallah et al. reported a non-invasive approach to endometriosis diagnosis based on saliva microRNA reverse PCR quantification combined with massive sequencing and an artificial intelligence algorithm [22].

In reproductive pathology, women with implantation failure have been shown to have elevated levels of Cf-DNA and circulating microRNA, and several studies have also demonstrated the importance of assessing Cf-DNA in various pathological conditions [23]. These include malignancy, inflammation, oxidative stress, infertility, and endometriosis. The Cf-DNA level in healthy individuals is variable, around 30 ng/mL (ranging from 0 to 100), and is regulated by a physiological endogenous endonuclease [24]. Many studies suggest that Cf-DNA found in healthy and diseased individuals is released after cell death via apoptosis [25]. Fragments of cell-free DNA may also originate from necrotic hematopoietic cells that are engulfed by macrophages and then partially released. DNA fragments released from necrotic cells are often much larger than apoptotic DNA fragments [25]. These mechanisms are not fully understood. According to this hypothesis, Cf-DNA levels should correlate with the extent of necrosis and/or cellular apoptosis.

In endometriosis, Zachariah et al. showed significantly higher nuclear and mitochondrial Cf-DNA in a group of 19 women with minimal to mild endometriosis compared to a control group (*p* = 0.046) [21]. The cut-off from an ROC curve showed a sensitivity of 70% and a specificity of 87%. The author concluded that Cf-DNA could be a potential biomarker for minimal and mild endometriosis diagnosis. More recently, Alonso et al. found no significant differences in any parameters of the Cf-DNA between women with and without endometriosis [26]. However, from the authors’ point of view, the low statistical power and heterogeneous pelvic pathology in the control group make it difficult to determine whether the negative results reflect a true lack of increase in circulating DNA in endometriosis. In 2022, Yuwono et al. reported that during menstruation and in endometriosis, the level of endometrial Cf-DNA was not altered [27].

The aim of our prospective, comparative, and controlled study is to quantify serum Cf-DNA levels in patients with and without endometriosis in combination with differential methylation status of candidate genes potentially involved in the pathophysiology of endometriosis.

## 2. Material and Methods

One hundred fifty-one patients were considered eligible because of a high suspicion of endometriosis based on clinical examination, pelvic ultrasound, and/or pelvic MRI performed to assess the degree of disease extension.

The inclusion criteria in our study were as follows: dysmenorrhea, pelvic pain, and a history of clinical examination, pelvic sonography, and/or MRI and/or laparoscopy with biopsies and cytological examination confirming the diagnosis of endometriosis. Confirmatory laparoscopy was performed or planned. Of the 151 women, 78 met the inclusion criteria, 38 of whom had proven endometriosis. Forty young women in the control group were selected because of a complete absence of symptoms (i.e., no known inflammatory disease, acute infectious syndrome, or pelvic or extra-pelvic pain).

**Figure 1:**
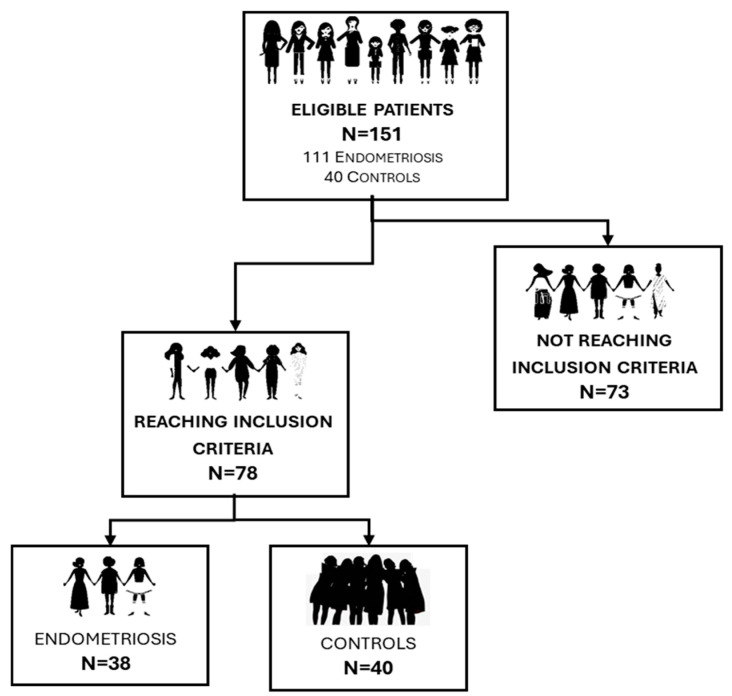
A group of 151 patients was selected based on age, genital activity, pelvic pain, and quality of life. In total, 78 met the inclusion criteria and a final 38 patients were classified as patients with endometriosis while 40 were taken as a control group.

Finally, seventy-eight patients between the ages of 20 and 40 were enrolled in our study: thirty-eight with endometriosis, pelvic pain, and/or reduced quality of life and a control group of forty healthy young women without pain or inflammatory disorder (Figure 1).

All patients duly signed a written informed consent form. All procedures were carried out under relevant guidelines and regulations. This study is part of our research project entitled the “ENDOFIV” protocol, approved by the Institutional Ethics Committee of our University Hospital on 10 February 2020 (ID-RCB number: 2019-A02858-49).

In the endometriosis group, the characteristics of the patients were equivalent to those of the control group in terms of age and BMI (see Table 1). One hundred percent of the participants with endometriosis suffer from pelvic pain, particularly dysmenorrhea. Seventy-eight percent of them suffered from mild endometriosis (stage I–II according to the classification of the American Society of Reproductive Medicine (ASRM)) and 22% from more severe endometriosis (stage III–IV). Moreover, 13% suffered from urinary problems (dysuria, pollakiuria), and none reported digestive issues.

### 2.1. Cf-DNA Extraction

Nucleic acids were extracted from 1 mL of frozen–thawed serum using a Qiagen QIAamp Circulating Nucleic Acid kit from Qiagen, Saint Quentin Fallavier, France, closely following the Handbook extraction protocol (10/2019). Briefly, 40 µL of proteinase K (concentration 600 mAU/mL) was added to the 1 mL of thawed serum. Then, 1 mL of lysis buffer containing 1.0 µg of carrier RNA (Qiagen ACL buffer) was added to the mix of the serum and proteinase K and vortexed for 30 s before incubation at 56 °C for 10 min. Then, 840 µL of ACB buffer was added to the lysate, mixed thoroughly by pulse-vortexing for 15–30 s, and incubated for 5 min on ice. The lysate–ACB buffer solution was carefully applied to the QiaAmp Mini column from Qiagen, Sant Quentin Fallavier, France to be drawn completely. After that, the column was washed and drawn successively with ACW1 and ACW2 buffers and ethanol (96–100%).

Then, the QIAamp mini-column was placed in a clean 2 mL collection tube and centrifuged at full speed (14,000 rpm) for 3 min, transferred to a new collection tube, and incubated for 10 min at 56° to be dried. Finally, cell-free DNA (Cf-DNA) was eluted twice with 25 µL of TE buffer (Tris/EDTA 1 mM/0.1 mM) using 1 min of full-speed centrifugation (14,000 rpm).

### 2.2. Cf-DNA Quantification via Reverse Real-Time PCR

Rpp30 (*NM_006413)* is commonly chosen to measure cfDNA levels in serum due to its role as a stable, single-copy housekeeping gene. As a robust internal control, *Rpp30* ensures accurate and reproducible Cf-DNA measurements. Rpp30 DNA (*NM_006413*) was quantified using qPCR genes from human serum samples.

This highly conserved endoribonuclease is present in all living cells in the body. Triplicates of 5 µL of Cf-DNA were added to 20 µL of PCR Light Cycler^®^ 480 SYBR Green I Master (Cat. no 04707516001) with 2.5 mM of MgCl_2_ and 0.5 mM of each forward and reverse RNase P primers (primer sequences: RNP30 Forward—AGATTTGGACCTGCGAGCG; RNP30 Reverse—GAAGCCGGGGCAACTCAC). A PCR product of 86 base pair regions spanning exon 1 and intron 2 of the Homo sapiens ribonuclease P/MRP subunit p30 gene (*NM_006413* and *ENST00000371703.7*) was obtained.

The amplification was performed on a Light Cycler 480 II (Roche) as follows: 35 cycles of 95 °C for 10 s, 59 °C for 20 s, and 72 °C for 15 s, followed by an elongation step of 5 min at 72 °C. Positive DNA controls at various concentrations and non-template controls were added to each run. Cycle threshold values were reported against a standard concentration curve, and Cf-DNA concentration was reported as the mean value of the triplicate.

#### Cf-DNA Methylation Analysis and Sequencing

Bisulfite DNA treatment was performed using an EZ DNA Methylation Kit (Zymo Research) following manufacturer recommendations. According to the initial concentrations, 35 µL of DNA sample was used for the reaction. At the end of the treatment, DNA was eluted with 25 µL of elution buffer and then diluted using 10 µL of H_2_O. Gene-specific PCR reactions were performed using Taq’Ozyme HS Mix (Ozyme, Saint-Cyr-l’École, France), 1 µL of bisulfite-treated DNA, and the final primers, each at a concentration of 0.4 µM, in a 20 µL final reaction volume using a C-100 thermocycler (Bio-Rad, Hercules, CA, USA). The cycling conditions were 95 °C/1 min; (95 °C/15 s–58 °C/15 s–72 °C/30 s) × 34; and 72 °C/5 s. All amplicon sizes were checked and validated via electrophoresis before sequencing, and sequencing was performed in the paired-end mode (2 × 150 bp) on the NextSeq Illumina Platform following the manufacturer’s protocol (BioProject record (NCBI): 1063938).

### 2.3. Statistical Analysis of the Rpp30 Quantification

Statistical analysis was performed with R software v.1. We first compared the quantification of RPP30 of the patient group with the control group with an ANOVA test.

### 2.4. Bioinformatic Data Analysis of the Methylation

A post-sequencing quality check was performed with the “FastQC” software (version 0.11.8), and sequence cleaning and paired-end read merging were performed using the “fastp” software (version 0.21.0). Then, a post-cleaning/merging quality check was performed with the “FastQC” software. The targeted gene sequences were extracted and sorted using custom “blast” software v.1. Finally, the frequency of C nucleotides versus the total number of C and T for each targeted position after bisulfite treatment was used to measure the rate of DNA methylation.

Methylated and unmethylated read counts were summed to calculate the total coverage at each CpG site, filtering out sites with fewer than 8 reads per sample or with constant methylation (fully methylated/unmethylated). Library sizes were normalized using the average total read count. A chi-square test of homogeneity was applied to each targeted sequence to assess differences in C and T nucleotide distributions between the control and endometriosis groups (alpha = 0.05). The analyses used R (4.3.1) and EdgeR (3.42).

### 2.5. In-House Classifier Model and Algorithm Used for Endometriosis Diagnostic

To build a classifier model for diagnostics, we developed an in-house algorithm linking Cf-DNA quantification and the level of methylation of nine specific genes that had given to the test these results:

We have designed and provided a sophisticated classification model enabled by testing five models, such as CART, logistic regression, SVM, random forest, and GLMnet. We finally chose the GLMnet (elastic net regularized generalized linear model) machine learning methodology, a statistical modeling technique used for regression tasks that had given the best overall results with our dataset

The best model was the one that used the GLMnet methodology, yielding the best AUC and accuracy over all the other methodologies, and adding later clinical variables will improve the prediction. It combines the L1 (Lasso) and L2 (Ridge) regularization penalties to achieve variable selection and handle multicollinearity, which is particularly useful when dealing with datasets with numerous predictors.

To obtain a robust predictor, the training and test sets were simulated 5000 times; these 5000 putative data sets were processed for benchmarking purposes.

We opted for a hold-out validation methodology, where the samples are randomized and then split into training and test data to emulate some variation in the training process; this allows for a strengthened prediction while reducing overtraining problems (in small samples when using machine learning models, they need to be optimized to avoid predicting features based on the bias of the entire sample).

Regularization is a key aspect of GLMnet, serving to prevent overfitting and improve model generalization. Lasso (L1 penalty) encourages sparsity in the model, effectively selecting important features by setting some coefficients to exactly zero.

Meanwhile, the Elastic Net combines L1 and L2 penalties, striking a balance between feature selection and regularization strength.

The process involves tuning hyperparameters, such as alpha and lambda, to optimize the trade-off between Lasso and Ridge regularization. Alpha determines the mix between the two penalties, with 1 corresponding to pure Lasso and 0 to pure Ridge. Lambda controls the overall strength of regularization, influencing the impact on the model’s coefficients.

We employ cross-validation to select the best combination of hyperparameters and to ensure the model’s robustness across different datasets. The resulting model is evaluated using metrics like accuracy, precision, recall, and accuracy score on a separate test set. The regularization path, visualizing the impact of regularization on each feature, aids in interpreting the model’s behavior.

To evaluate the performance of our tool, we compared all the AUC (area under the curve), specificity (focuses on true positives and minimizes false negatives, i.e., patients with endometriosis), sensitivity (focuses on true negatives and minimizes false positives, i.e., patients without endometriosis), and accuracy (overall correctness for the correct identification of positive cases and negative cases) of all simulations and retained the best predicting model.

The model’s final behavior step is to compute a linear combination of the predictors retained as a score or INDEX, which allows the prediction of the status (with or without endometriosis).

The final formula to predict the patient’s status has been set as follows: INDEX (the prediction of the patient’s status). EQUAL (9 variables representing the respective genes’ methylation percentages and an RPP30 copy number) MULTIPLIED (by the respective beta coefficients associated with each gene).

## 3. Results

Seventy-eight women were enrolled in the study. Of the 78 women, 38 had endometriosis, and 40 healthy women formed a control group.

### 3.1. Rpp30 QPCR Absolute Quantification

To quantify the absolute amount of the Hs_*Rpp30 gene* in a 1 mL serum sample, we calculated a standard curve using a plasmid containing a portion of the *Rpp30 gene*, a single-copy gene present in the human genome. The *Hs_Rpp30*-positive control (IDT, ref. 10006626) is calibrated at a concentration of 200,000 copies/µL in Tris/EDTA at a pH of 8.0.

**Figure 2:**
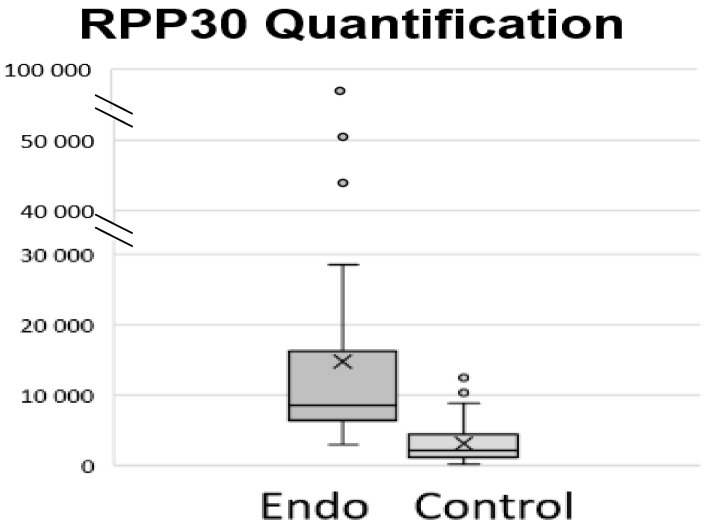
The box plots represent the median, interquartile range, minimum, and maximum values of RPP30 gene expression in copy number for the cohort of 78 samples. There is a significant difference in the RPP30 copy numbers between the two groups: The statistical analysis was performed using an ANOVA test (*p* = 7.63 × 10^−5^).

The absolute quantification and mean absolute quantification between the control and endometriosis groups of Rpp30 genes are shown in Figure 2 and Table 2.

### 3.2. Targeted Genes and Differential Methylation Analysis

In an earlier proof-of-concept study, we identified 15 candidate genes involved in cell cycle regulation, DNA reparation, and oxidative stress rescue with different methylation profiles after whole-genome sequencing (WGS). In this group of genes, 10 were hypermethylated and 5 were hypomethylated.

In the present study, after gene depletion and selection, we analyzed the differential methylation status of nine targeted genes (*CALD1*, *RRP1*, *FN1*, *DIP2C*, *RMI2*, *TDRD5*, *USP1*, *HDAC1*, and *DNMT1*) for which the differential methylation differed between the two groups (see the details in Table 3).

The *p*-values indicate that four genes (DIP2C, DNMT1, RRP1, and USP1) are significantly differentially hypomethylated in the endometriosis group compared to the control group.

**Figure 3:**
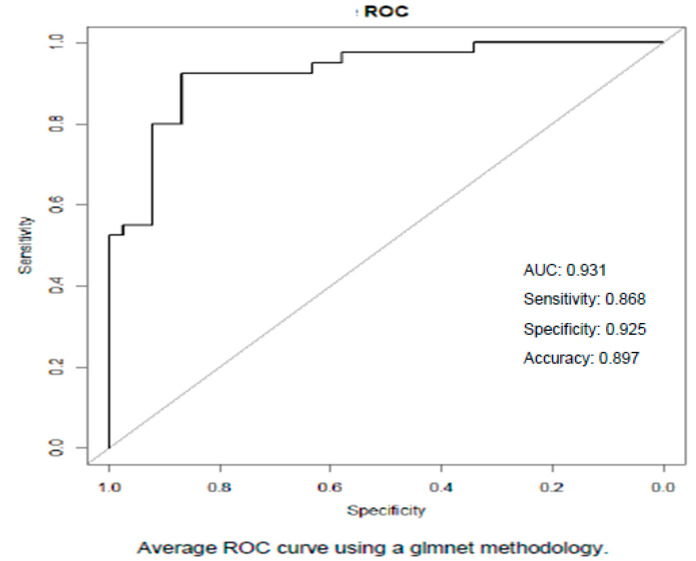
Average ROC curve using data from Glmnet methodology.

The *p*-value for *TDRD5* is close to the significant threshold, and *DNMT1* was widely expressed. The use of the algorithm linking Cf-DNA quantification and the methylation status of nine specific genes gave an AUC (area under the curve) of 93.1%, a sensitivity of 86.8%, a specificity of 92.5%, and an accuracy of 89.7% between the two groups after real blind testing of the samples (Figure 3).

## 4. Discussion

Many strategies for the identification of biomarkers to prevent and detect endometriosis, such as response markers to systemic inflammation, natural killer cells, lymphocyte cell typing and dysregulation, and the proteome of ectopic endometrium, are proposed [28]. Recently, Omes and Omics methods have been reported in the literature as innovative options for endometriosis diagnosis. Azeze et al. reported that proteome profiles from various noninvasive clinical samples collected in women suffering from endometriosis were significantly differentially expressed compared to women without endometriosis [29].

At the genetic level, a specific signature of necroptosis-related genes was reported by Wang et al. [30], and a specific model analysis reported seven specific genes [31] as diagnostic markers of endometriosis. Endometrial single-cell ribonucleic acid sequencing (scRNA-seq) [32] and piwi RNA saliva-reverse transcription and sequencing [33] were proposed as diagnostic signatures for endometriosis.

Our study, which proposes an innovative and non-invasive method, is, to the best of our knowledge, the only controlled, prospective, and comparative study demonstrating high levels of Cf-DNA with an epigenetic differential methylation signature of four genes in the serum of women with pelvic pain and endometriosis.

The four hypomethylated genes in endometriosis are *RRP1*, *DIPC2*, *USP1*, and *DNMT1*. Among these, USP1 exhibits the highest degree of hypomethylation, followed by the *DIP2C* and DBNT1 genes, suggesting the most substantial upregulation in gene expression.

*USP1* (ubiquitin-specific protease 1) encodes an enzyme that belongs to the deubiquitinase family. Deubiquitinases modify target proteins by removing ubiquitin chains. This enzymatic activity plays a crucial role in post-translational protein regulation, impacting numerous cellular processes, including gene methylation and activation. Similarly, the *DIP2C* gene triggers substantial DNA methylation and gene expression changes.

Both the *USP1* and *DIP2C* genes can influence the ubiquitination state of histones, such as H2A or H2B. This is critical for chromatin remodeling, which can either facilitate or inhibit access of DNA methyltransferases (DNMTs) or other methyltransferases. In endometriosis, inflammatory processes may be aberrantly activated due to the overactivity of hypomethylated genes. To our knowledge, no studies have yet explored the relationship between the activity of these four genes and endometriosis, beyond the established involvement of epigenetics in endometriosis via *DNMTs*.

Cell-free DNA from peripheral blood was chosen as a biomarker of cell death and pathophysiology signals and because it is easy to extract, quantify, and sequence [24]. In addition, targeted genes for endometriosis were identified, and their methylation status was assessed.

Combining Cf-DNA quantification and the differential methylation assessment of a specific group of targeted genes creates interesting opportunities for an early diagnosis of the disease. Cf-DNA is one of the most interesting classes of human molecules. Studies on Cf-DNA have led to numerous innovations in many fields, particularly in oncology, expanding the possibilities for the preventive and predictive noninvasive diagnosis and prognosis of different pathologies and diseases. The mechanism by which Cf-DNA is released into the bloodstream is still poorly understood. However, it is known that Cf-DNA in peripheral blood is released after cell death via apoptosis or necrosis [34], mainly from hematopoietic cells in healthy individuals and from diseased tissue. Necrosis and cell apoptosis determine the number of Cf-DNA fragments released into the bloodstream, either actively or passively. In the present study, there was a significant difference in the RPP30 copy number between the two groups. On average, there are 3.9 times as many copies of the RPP30 gene in the endometriosis group as there are in the control group.

Cf-DNA contains the same genetic and epigenetic variations as nuclear and mitochondrial DNA from viable cells. These variations are copy number variations, mutations, Cf-DNA compositions, and methylations. As such, Cf-DNA reflects a patient’s genetic profile. In excess, Cf-DNA in serum is also indicative of oxidative stress, potentially systemic inflammation, and/or infection, which is associated with numerous pathologies, including endometriosis, making it a new potential biomarker and genetic assay in ART [35].

Speculating on the inflammation and pain caused by the disease, we extracted Cf-DNA from thirty-eight young, sexually active women with pelvic pain and endometriosis. The levels of Cf-DNA were found to be at least four times higher among these women than in a control group of women without endometriosis.

The discrepancies observed in some studies evaluating the levels of Cf-DNA in serum are due to the difficulty of comparing them with poorly identified control subjects. Serum Cf-DNA levels must be interpreted with caution, as their elevation may be due to pathologies other than endometriosis relating to lifestyle and a variety of biological changes. Therefore, measuring Cf-DNA alone is not sufficient to make a diagnosis of endometriosis. The epigenetic characterization of Cf-DNA enhances its potential as an early marker.

In an earlier unpublished study, we performed Cf-DNA genome sequencing on samples from 10 patients. We identified 62 targeted gene candidates for endometriosis whose methylation status allowed us to extract 15 genes, of which 10 were hypermethylated and 5 were hypomethylated. All genes identified in our study were included in the list of most frequently cited genes in endometriosis based on cellular DNA studies.

Of the 15 genes (CALD1, RRP1, FN1, FAML87B, TCEAL6, RPL29P2, ATP11A-AS1, DIP2C, SLCO2B1, RMI2, FBX038, ACVR2A, USP1, TDRD5, and STAU2-AS1), we kept eight for further study and added DNMT1 (DNA methyltransferase 1) to control the methylation process.

Methyl groups are positioned on DNA and change the way the cell reads the genome. The presence of methylated cytosines in the promoter sequence of a gene is associated with transcriptional repression, as it directly inhibits the binding of transcription factors and can also recruit repressors from the MBP (methyl-binding protein) family. Since methylation profiles are faithfully copied during genome replication through the action of the enzyme *DNMT1* (DNA methyltransferase 1), DNA methylation can be seen as an epigenetic memory system that stably maintains the repressed state of certain genes in a cell population.

In our study, four genes were hypomethylated. This meant that they were highly expressed regardless of the stage of endometriosis.

These findings are very promising in terms of their robustness and merit further investigation. The proteins encoded by one of our differentially methylated genes are involved in the final stages of nucleogenesis. Another is expressed in the nervous system. A third is a potential diagnostic and prognostic factor associated with cell proliferation. The last plays a role in the progression of several tumors.

The observed hypomethylation of these genes not only indicates an early expression potential in endometriosis but also positions them as robust markers of the disease. Screening for these four genes is proving to be a reliable method for identifying endometriosis and providing crucial insights into their evolutionary dynamics.

Of particular note is the role of *CALD1* (calmodulin), a much-cited gene in endometriosis research. Our investigation of cellular DNA revealed its involvement in promoting cell migration, vascularization, invasion, proliferation, and general cellular mobility. In our initial study, we identified significant calmodulin hypermethylation in the serum of women with endometriosis. Although this initial investigation focused on a single CpG, our more recent results take a broader perspective. It is also interesting to note that our approach differs from those using panels of post-transcriptional miRNAs; it is less costly (i.e., does not require sequencing) and it is easier to set up in molecular biology laboratories using real-time PCR.

In summary, our landmark study demonstrates for the first time a substantial increase in free circulating DNA associated with four candidate genes that show significant differential methylation in the serum of women with endometriosis.

## 5. Future Perspective

This distinctive molecular signature, based on free circulating DNA quantification and differential methylation analysis of a specific group of genes, opens a new potential of predictive and preventive diagnosis of endometriosis. Also, we believe that any clinical laboratory medicine, with good basic molecular biology experience, can offer the test at a very competitive cost, with a short turnaround time (TAT), to obtain an informative result.

## Figures and Tables

**Table 1:** Patient characteristics. BMI: body mass index; VAS: visual analog scale.

Groups	Endometriosis *n* = 38		Controls *n* = 40
Mean age	25.5 (range 22–45)		24 (22–26)
BMI (mean)	23.7 (range 21–28)		25.2 (20–27)
History			
Dysmenorrhea	38 (100%)		0
Infertility	17 (45%)		-
ASRM classification	St. I–II	30 (78%)	-
	St. III–IV	8 (22%)	
Pelvic pain outside	2 (5%)		0
Pain (VAS mean)	6.5 (5.5–9)		-
Urinary disorders	5 (13%)		0
Digestive disorders	-		-

**Table 2:** Absolute quantification of the cohort of 78 patients. Cf-DNA concentration in serum was much higher in the endometriosis group than in the control.

	RPP30 Copy Number			
	Mean	Median	Max	Min
Endometriosis	14,762	8602	96,981	2927
Control	3166	2224	12,498	161
Difference	11,596	6378		
Factor	4.7	3.9		

**Table 3:** Details and functions of the nine genes and associated *p*-values.

HUGO Name	GENE Name	Summary	*p*-Value
CALD1	Caldesmon 1 (Testis Secretory Sperm-Binding Protein Li 227n)	This gene encodes a calmodulin- and actin-binding protein that plays an essential role in the regulation of smooth muscle and nonmuscle contraction.	0.4009
RRP1	Ribosomal RNA Processing 1	This protein coding gene. Among its related pathways are rRNA processing in the nucleus and cytosol and processing of capped intron-containing pre-mRNA.	2.83 × 10^−3^
FN1	Epididymis Secretory Sperm Binding Protein	This gene encodes fibronectin, a glycoprotein present in a soluble dimeric form in plasma, and in a dimeric or multimeric form at the cell surface and in extracellular matrix. The encoded preproprotein is proteolytically processed to generate the mature protein. Fibronectin is involved in cell adhesion and migration processes including embryogenesis, wound healing, blood coagulation, host defense, and metastasis.	0.9328
DIP2C	Disco Interacting Protein 2 Homolog C	DIP2C gene triggers substantial DNA methylation and gene expression changes. Particularly, it modulates expression of the PALB2 gene involved in homologous recombination repair and the DMAP1 gene, the DNA methyltransferase 1-associated protein 1 involved in transcription repression and activation.	2.22 × 10^−5^
RMI2	RecQ-Mediated Genome Instability 2	An essential component of the RMI complex and a complex that plays an important role in the processing of homologous recombination intermediates.	0.11
TDRD5	Tudor Domain-Containing 5	Predicted to be involved in DNA methylation involved in gamete generation;	0.846
USP1	Ubiquitin Specific Peptidase 1	This gene encodes a member of the ubiquitin-specific processing (UBP) family of proteases that is a deubiquitinating enzyme (DUB) with His and Cys domains.	2.15 × 10^−15^
HDAC1	Histone Deacetylase 1	Histone acetylation and deacetylation, catalyzed by multisubunit complexes, play a key role in the regulation of eukaryotic gene expression. The protein encoded by this gene belongs to the histone deacetylase/acuc/apha family and is a component of the histone deacetylase complex.	0.2761
DNMT1	DNA Methyltransferase 1	This gene encodes an enzyme that transfers methyl groups to cytosine nucleotides of genomic DNA. This protein is the major enzyme responsible for maintaining methylation patterns following DNA replication and shows a preference for hemi-methylated DNA.	1.05 × 10^−4^

HUGO: HGNC human gene nomenclature (https://www.genenames.org/). Summary sources: GeneCards (https://www.genecards.org/).

## Data Availability

The datasets generated and analyzed during the current study are available in the NCBI Bio Project (Bethesda, MD, USA), http://www.ncbi.nlm.nih.gov/bioproject/1063938 (accessed on 28 October 2024).
